# Associated Factors of Pneumonia in Individuals with Chronic Obstructive Pulmonary Disease (COPD) Apart from the Use of Inhaled Corticosteroids

**DOI:** 10.3390/biomedicines11051243

**Published:** 2023-04-22

**Authors:** Rosario Lineros, Lourdes Fernández-Delgado, Antonio Vega-Rioja, Pedro Chacón, Bouchra Doukkali, Javier Monteseirin, David Ribas-Pérez

**Affiliations:** 1 Unidad Salud Mental, Hospital Vázquez Díaz, 21080 Huelva, Spain; 2 UGC de Alergología, Hospital Universitario Virgen Macarena, 41009 Sevilla, Spain; 3 Hospital Quirón Sagrado Corazón, 41013 Sevilla, Spain; 4 Department of Stomatology, Faculty of Dentistry, University of Seville, 41004 Seville, Spain

**Keywords:** COPD, pneumonia, inhaled corticosteroids

## Abstract

Inhaled corticosteroids (ICSs) are widely used in chronic obstructive pulmonary disease (COPD) and in combination with long-acting β2 agonists (LABAs) to reduce exacerbations and improve patient lung function and quality of life. However, ICSs have been associated with an increased risk of pneumonia in individuals with COPD, although the magnitude of this risk remains unclear. Therefore, it is difficult to make informed clinical decisions that balance the benefits and adverse effects of ICSs in people with COPD. There may be other causes of pneumonia in patients with COPD, and these causes are not always considered in studies on the risks of using ICSs in COPD. We consider it very useful to clarify these aspects in assessing the influence of ICSs on the incidence of pneumonia and their role in the treatment of COPD. This issue has important implications for current practice and the evaluation and management of COPD, since COPD patients may benefit from specific ICS-based treatment strategies. Many of the potential causes of pneumonia in patients with COPD can act synergistically, so they can be included in more than one section.

## 1. Introduction

### 1.1. Pneumonia in Patients with COPD Independent of the Use of ICS

Although pneumonia is often seen as a potential side effect of inhaled corticosteroids (ICSs) in individuals with chronic obstructive pulmonary disease (COPD), the actual frequency of pneumonia varies across studies, and certain analyses have reported varying rates of pneumonia associated with different types of ICS treatments [1,2,3].

Several randomized controlled trials (RCTs) that involved a significant number of participants did not demonstrate an increase in the occurrence of pneumonia in patients who were randomly assigned to undergo ICS treatment [4,5,6]. These findings imply that other factors, which are not related to the utilization of ICSs, could impact the probability of developing pneumonia in individuals with COPD.

### 1.2. Definition of COPD and Pneumonia

The term COPD may not be precise in distinguishing various pulmonary conditions, including asthma, which can pose difficulties in distinguishing asthma from COPD, particularly among smokers and older adults. Moreover, some patients may exhibit clinical features that are common to both asthma and COPD [7]. The diagnosis of pneumonia based solely on clinical signs and laboratory data can be challenging, given the variability of clinical presentations, particularly in the presence of chronic respiratory illness. The literature on COPD often lacks clarity in defining, documenting, classifying, and attributing pneumonia events in terms of severity. Furthermore, variations in the methods used to document and evaluate pneumonia across different countries can contribute to the variability in reported incidence rates, making cross-trial comparisons problematic. Patients with severe forms of COPD may experience exacerbations that can be challenging to differentiate clinically and radiographically from pneumonia, resulting in an overestimation of the number of pneumonia cases in these trials [8].

Patients with acute exacerbations of COPD (AECOPD), a serious public health issue, may exhibit symptoms resembling pneumonia.

Clinically relevant pneumonia can be differentiated from other comparable illnesses using traditional diagnostic criteria including a differential blood count and C-reactive protein (CRP) levels. However, such methods have suboptimal sensitivity and specificity in patients with suspected infections, leading to uncertainty when starting treatment [9].

Bacterial colonization has been linked to increased inflammation, symptom aggravation, and more frequent exacerbations. Nearly 44% of all samples collected from stable COPD patients tested positive for bacterial colonization [10].

## 2. Various Aspects of Infection in COPD and Its Capacity to Generate Pneumonia

It is widely acknowledged that potentially harmful microorganisms can inhabit the bronchial area in individuals with COPD. Studies have indicated that the composition of the lung microbiome in patients with stable COPD differs significantly from that of healthy individuals [11,12,13,14,15,16,17,18]. There is evidence of a relationship between the appearance of exacerbation symptoms and the acquisition of new bacterial strains [19]. The majority of information about COPD is obtained from samples collected through various methods such as biopsies, lung tissue explants, bronchoalveolar lavage (BAL), protected specimen brush (PSB) techniques, and sputum. Research studies have shown that sputum samples contain different types of microorganisms compared to bronchoalveolar samples, and it has been confirmed that the lungs of COPD patients have unique microbiomes that differ from healthy individuals in terms of bacterial composition [20,21]. Alterations in the microbiome of the upper or lower respiratory tract can affect the immune response and make the host more vulnerable to developing pneumonia [22].

Antibiotic resistance in bacteria that cause community-acquired pneumonia (CAP) such as pneumococci and Mycoplasma pneumoniae is becoming more common, which is a significant global health concern and a contributing factor in the increasing burden of disease, particularly in COPD patients. This information has been reported by several studies, including references [23,24,25].

Pathogens have several ways of developing resistance to antibiotics, such as producing beta-lactamase, losing outer membrane proteins that are susceptible to antibiotics, changing their targets, forming biofilms, using efflux pumps, and acquiring integrons. The use of antibiotics at low concentrations can also contribute to the development of resistance among the exposed pathogens. Apart from the misuse of antibiotics in healthcare, the general population is exposed to non-iatrogenic antibacterial drugs in their daily lives, such as antibiotics used in the meat industry to treat livestock, leading to an increase in drug resistance in pathogens. The increased use of antibiotics in both clinical and nonclinical settings has resulted in an increasing number of clinically isolated drug-resistant strains, including carbapenem-resistant Klebsiella pneumoniae [26].

The symptoms of SARS-CoV-2 infection can vary widely, from showing no symptoms at all to developing atypical pneumonia. COVID-19 pneumonia is a disease that continues to evolve over time. In fact, around 40% of cases of SARS-CoV-2 infection do not display any symptoms, another 40% have mild upper respiratory symptoms, and approximately 20% develop pneumonia [27,28,29].

Klebsiella species consist of *Klebsiella ozaenae, Klebsiella rhinoscleroma*, and *Klebsiella pneumoniae*, with the latter having significant clinical implications as an important opportunistic and iatrogenic infectious pathogen. *K. pneumoniae* typically colonize different mucosal surfaces, including the upper respiratory tract, in humans. They are a significant cause of respiratory tract infections that can result in severe pneumonia and are transmitted to the human body via contaminated respirators, atomizers, catheters, and through self-contamination by colonizing bacteria.

The immunological system’s growth and maintenance, as well as general health, depend significantly on microflora. Dysbiosis, or changes in the microbial makeup and function in the intestinal and respiratory tract, has recently been connected to variations in immune responses and pulmonary development. Chronic gastrointestinal tract (GIT) disorders such as inflammatory bowel disease (IBD) or irritable bowel syndrome (IBS) frequently coexist with chronic lung diseases such as asthmatic processes or COPD.

Adults with IBD (up to 50%) and IBS (up to 33%) may exhibit symptoms of pulmonary inflammation or compromised pulmonary function. IBD diagnoses are also more common in COPD patients.

In patients with COPD, the intestinal lining is more permeable than in healthy individuals. Although the GIT and respiratory tract have different functions and environments, they share a common embryonic origin and structural similarities. Thus, it is not surprising that they can interact in both health and disease [30,31,32,33,34].

Research indicates that commensal microbes in the respiratory tract may contribute to the development of pneumonia. Furthermore, studies have shown that the intestinal microbiota play a crucial role in regulating local and systemic host responses in clinically relevant models of pneumonia. The intestinal microbiota have been shown to offer protection against pneumonia by priming alveolar macrophages. The interaction between the intestine and lungs during infection has been observed, and it has been suggested that the intestinal microbiota can modulate the immune response in the lungs [35,36].

Periodontal diseases are a group of infectious conditions caused by multiple microorganisms, such as gingivitis and periodontitis, that affect the tissues supporting the teeth. These diseases have been associated with the worsening of various respiratory diseases. The presence of pathogens in the mouth can directly enter the lungs, increasing the risk of acute exacerbations of chronic obstructive pulmonary disease (AECOPD) or community-acquired pneumonia (CAP) in individuals with COPD [37].

**Figure 1:**
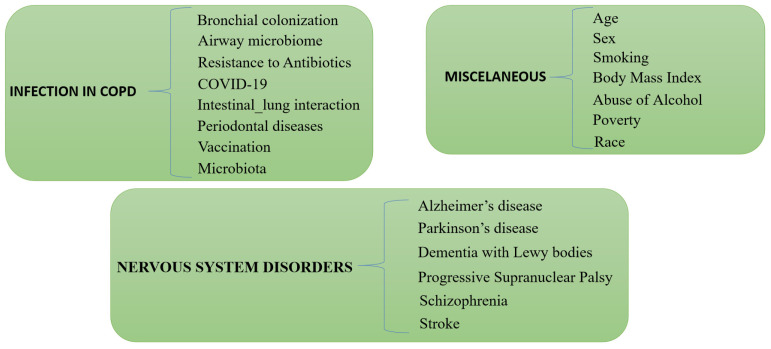
Infections in COPD, miscellaneous, and nervous system disorders as causes of pneumonias in COPD independent of the use of ICS.

Vaccination against influenza, as well as Pneumococcus pneumoniae, has been proven to be effective in preventing infections not only in patients with COPD but also in individuals who are in close contact with them, thus reducing the risk of transmission. Encouraging complete vaccination can contribute to better healthcare management and allocation for patients with COPD [38,39] (Figure 1).

### 2.1. Miscellaneous: Age, Sex, Body Mass Index, Smoking, and Social Status

The elderly are more likely to develop pneumonia due to decreased organ function and an aging immune system, and may suffer multiple organ dysfunction syndrome (MODS), respiratory and circulatory failure, and even death. In fact, pneumonia has become one of the leading causes of death in the elderly [40].

The likelihood of developing community-acquired pneumonia (CAP) depends on age and underlying medical conditions, with older individuals aged 65 or above who have COPD being at a higher risk. Within the COPD population, those aged 65–79 or over 80 are more susceptible to CAP than those aged 45–65. Several independent factors have been identified for recurrent CAP in adults, including COPD, older age, and a lack of pneumococcal vaccination [6,40,41,42,43,44].

The impact of seasonal influenza on health varies based on age and pre-existing medical conditions. Individuals aged 65 or older with COPD are at higher risk of serious medical complications leading to hospitalization and death. COPD is the third leading cause of death among people aged 65 and above in the United States. About 15 million adults in the US have been diagnosed with COPD, and 5 million of them are aged 65 or older. For people with COPD, influenza infection can cause severe complications and even death, especially in older adults. Patients with COPD have a higher risk of respiratory failure and are more likely to exhibit frailty. Any respiratory infection, including influenza, can cause airway inflammation and constriction, making it difficult to breathe properly. This inflammation can increase the likelihood of COPD exacerbations. As a result, people with COPD are advised to receive seasonal influenza vaccination [45].

Adjusted incidences of all types of pneumonia, including community-acquired pneumonia (CAP) and hospital-acquired pneumonia (HAP), are higher in men than in women. Specifically, men have a 47% higher adjusted incidence of CAP and a 98% higher adjusted incidence of HAP than women. Furthermore, men with COPD have a higher risk of developing pneumonia than women with COPD [46].

A single predictive factor for COPD is body mass index (BMI), and there is a direct correlation between lower body weight and higher death rates in COPD patients. While evidence suggest that people who can acquire weight have a better prognosis, more weight loss increases the danger. The prognostic relevance of a low BMI could be the result of waste in end-stage COPD. In turn, underweight patients may be less resistant to infections such as pneumonia [47,48,49].

Additionally, smoking has been recognized as a risk factor for CAP, with the likelihood altering depending on the history of smoking. Smoking’s impact on the risk of CAP has been studied. Smoking was verified as an independent risk factor in the multivariate analysis, and both current smokers and former smokers were shown to have a greater risk of CAP compared to non-smokers. With higher pack years, the risk grew higher. The risk of CAP was substantially greater in ex-smokers who had quit smoking more recently (4 years) compared to those who had quit smoking less recently. Those who had never smoked but were exposed to passive smoking were similarly at a significantly higher risk of CAP. Likewise, elderly individuals aged 65 years or older who were subjected to involuntary exposure to passive smoke within their domestic environment exhibited a heightened susceptibility to community-acquired pneumonia (CAP). It has been observed that individuals who are current smokers and aged 65 years or above, are at a greater risk of contracting the infection compared to those who are ex-smokers, regardless of their age [4,6,50,51,52,53,54,55].

According to the Centers for Disease Control and Prevention (CDC), people who abuse alcohol are 10 times more likely to develop pneumococcal pneumonia and 4 times more likely to die from pneumonia than non-drinkers [56,57]. Heavy drinkers are more likely to suffer from COPD according to the Global Initiative for Chronic Obstructive Lung Disease (GOLD) criteria and suffer more respiratory symptoms [58].

Poverty is associated with an increased risk of pneumonia [59]. Although race is a characteristic that must be taken into account, it can be conditioned by other co-existing factors such as socioeconomic condition. For example, Black adults in the United States are 2.6 times more likely to develop pneumonia than Caucasian adults, although in general, the former are also 4.4 times poorer than Caucasian adults [60,61] (Figure 1).

### 2.2. Nervous System Disorders

Advanced-stage Alzheimer’s disease (AD) is often accompanied by two serious medical conditions: dysphagia and aspiration pneumonia. Dysphagia associated with pseudobulbar palsy can cause weight loss, despite attempts to manage swallowing difficulties. The underlying mechanisms that regulate the body’s basic functions, such as maintaining nutritional status, appear to be impaired in these patients. Aspiration pneumonia is the leading cause of death in end-stage AD.

Aspiration pneumonia is caused by a number of factors such as dysphagia, reduced consciousness, a loss of gag reflex, periodontal disease, and the mechanical effects of inserting tubes into the respiratory and gastrointestinal tracts. The bacteria responsible for the infection include oral flora, mostly aerobes, and nosocomial-acquired pathogens, such as Staphylococcus aureus, aerobic and facultative Gram-negative bacilli, which may colonize patients in hospitals and nursing homes. Besides the use of antibiotics, addressing the symptoms and managing AD in patients with pneumonia is critical in alleviating their suffering [32].

Neurological disorders such as Alzheimer’s disease, Parkinson’s disease, Lewy bodies, and progressive supranuclear palsy have been found to increase the risk of pneumonia that is confirmed through autopsy. Patients with these conditions are often bedridden and may experience dysphagia, altered mental states, or respiratory muscle weakness, all of which can increase the risk of pneumonia. Additionally, the causes of pneumonia in patients with neurological conditions who also have COPD may be different from those in the general population, which can lead to differences in the distribution of pathogens causing the infection [62].

Patients with schizophrenia are at increased risk of developing pneumonia, as well as other respiratory diseases such as COPD and asthma [63].

HAD is one of the most important complications that can be prevented in a patient who has suffered a stroke. The occurrence of pneumonia worsens the prognosis of a patient, especially if he or she has had an ischemic stroke, reducing the chances of a full recovery and increasing the chances of bodily deterioration that can lead to the patient’s death. Although the chances of developing pneumonia in the general population range from approximately 8% to 30%, it increases to 44% in patients with COPD and who have had a stroke [64] (Figure 1)

## 3. Hospitalization and Similar Conditions

Hospital-acquired pneumonia (HAP) is a type of infection that patients acquire during their hospital stay. It is a common nosocomial infection that causes significant clinical and economic burdens, including prolonged hospitalization, high medical costs, and increased morbidity and mortality.

Hospitalization alone can increase the risk of pneumonia, but there are additional factors associated with hospitalization that can further increase this risk. These factors include older age, male gender, pre-existing asthma, pre-existing COPD, pre-existing chronic lower airway disease, tube feeding, suctioning, positioning, use of mechanical ventilation, admission to the ICU, poverty, race, anemia, dementia, chronic kidney disease, paraplegia, hemiplegia, and metastatic carcinoma. These factors may differ from those seen in the general population and may increase the risk of pneumonia in hospitalized patients.

− Factors specific to hospitalization.
− Type of hospital: It has been reported that the incidence of hospital-acquired pneumonia (HAP) in tertiary hospitals is lower than in general hospitals. Specifically, the incidence in tertiary hospitals is around 3.5%, while in general hospitals, it is around 5.7%. This difference could be attributed to variations in the quality of patient care and access to healthcare resources between the two types of hospitals. Tertiary hospitals may have better environmental hygiene practices and more highly trained healthcare professionals, which could lead to better quality care and ultimately a lower incidence of HAP.
− Type of patient: The prevalence of HAP is relatively higher among medical patients compared to surgical patients. Nevertheless, it is important to acknowledge that surgical patients remain susceptible to the onset of HAP.
− Higher bed-to-nurse ratios: Patients who are hospitalized in facilities that exhibit bed-to-nurse ratios rated as g 4 and 5 experience a 1.4-fold elevated risk of HAP in comparison to those under the care of hospitals that maintain a bed-to-nurse ratio classified as grade 1. From a practical standpoint, nurses who are assigned a reduced patient load would be in a position to allocate more time and resources towards the provision of care to those patients who are entrusted under their purview.
− Type of room: The incidence of HAP exhibits a notable approximately threefold increase in patients whose accommodation comprises more than four beds, compared to those who are placed in rooms containing three or less beds. Patients who are accommodated in individual patient rooms are likely to experience a reduced risk of HAP compared to their counterparts residing in multi-patient rooms. The mitigation of HAP risk can be attributed to the limited exposure of patients in single-patient rooms to potential reservoirs, and attributed to limited or no direct and indirect contacts [58,59,60,61,62,63].

Aspiration pneumonia (AP) is an infectious condition caused by inhalation of oropharyngeal secretions populated by pathogenic bacteria, whereas aspiration pneumonitis (Mendelson syndrome) (MS) is a chemical injury brought on by the inhalation of sterile gastric contents.

**Figure 2:**
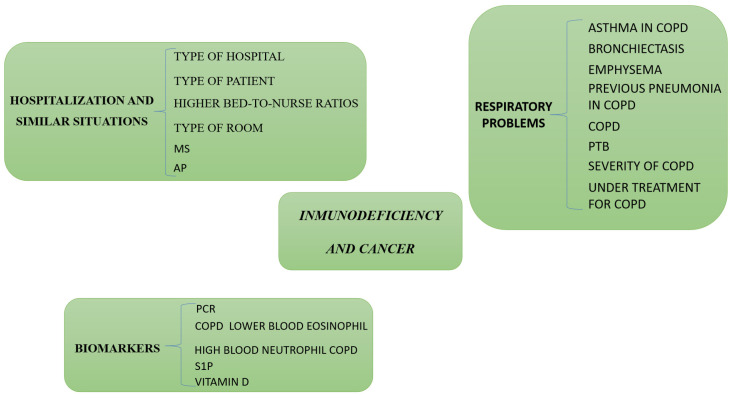
Hospitalization and similar situations, respiratory problems, immunodeficiencies, and cancer as causes of pneumonias in COPD independent of ICS use. PCR: C-reactive protein. S1P: Sphingosine-1-phosphate. NLR: neutrophil–lymphocyte ratio. AP: Aspiration pneumonia. MS: Mendelson syndrome. PTB: Pulmonary tuberculosis.

Factors predisposing to MS and AP include a decreased level of consciousness, neurological disorders, dysphagia, and material aspiration in association with tracheostomy [37,38] (Figure 2).

### 3.1. Respiratory Problems

The presence of asthma has been identified as an independent risk factor for pneumonia in the population of COPD and has been associated with a 13% risk of pneumonia [39]. Asthma has been shown to be the strongest independent risk factor for pneumonia in patients with COPD, resulting in more exacerbations and more severe limitation of airflow compared with patients with COPD alone [16].

Structural changes in COPD, such as bronchiectasis, can modulate the severity of the exacerbation and contribute to the morbidity associated with the latter. In 2014, GOLD first described bronchiectasis as one of the comorbidities of COPD [42].

Bronchiectasis and COPD are found to coexist in 20–60% of all cases. However, patients with severe COPD are reported to have a particularly high frequency of bronchiectasis, although the published figures vary widely between 4 and 72%. In patients with COPD and bronchiectasis, the main clinical manifestations are those typically associated with bronchiectasis: chronic cough and sputum production, chronic bronchial infection, and frequent infective exacerbations [43,44].

Computed tomography (CT)-diagnosed emphysema has been identified as the strongest risk factor for pneumonia among all clinical parameters in patients with COPD. The presence of emphysema has been associated with the severity of pneumonia in patients with COPD. Lower lobe emphysema is particularly associated with the frequency of exacerbation in COPD, according to the involvement of lung structures such as diameters [52,53].

An important factor that can be a predisposition to pneumonia in COPD is a history of previous pneumonia episodes. Patients with COPD who have suffered pneumonia before have an increase incidence of CAP [6,16].

Patients with previous COPD are at increased risk of developing pneumonia compared to otherwise healthy individuals and often have poorer clinical outcomes in terms of the severity of the pneumonia [16].

*Mycobacterium tuberculosis* has the ability to infiltrate a multitude of body organs. Pulmonary tuberculosis (PTB) frequently manifests as the predominant clinical form of the disease, primarily characterized by pulmonary involvement resulting in respiratory impairment. This ailment is a common chronic consumptive illness; however, it may manifest itself as an acute pneumonia. Acute tuberculous pneumonia (TP) bears resemblance to conventional bacterial pneumonia in its clinical presentation. Acute TP is commonly associated with the clinical presentations of CAP; however, in the present scenario, the etiological agent is microplasma. Tuberculosis should be preferred over non-tuberculous bacteria or viruses. In developing nations, *M. Tuberculosis* serves as the primary etiological agent responsible for the onset of community-acquired pneumonia (CAP). On the other hand, the relationship between tuberculosis and COPD is well known [65,66].

Several studies have shown that an increase in infections, especially respiratory infections, increases the risk of developing pneumonia [36,67,68].

The co-occurrence of inflammatory diseases in both the upper and lower respiratory tracts is a common phenomenon. Sino-pulmonary disease is a widely acknowledged medical condition, particularly in cases where the ailment has progressed to a chronic stage. Individuals who exhibit significant sphenoid involvement, characterized by either complete or partial opacification, possess a heightened likelihood of being diagnosed with CAP, with a relative risk of 20 times that of individuals without such involvement [69].

Significant differences in the risk of pneumonia have been reported according to the severity of COPD. The severity of the underlying respiratory disease affects the risk of CAP. Studies on individuals aged 65 years and above with mild lung disease, i.e., not necessitating medication or oxygen, have indicated a twofold increase in the likelihood of contracting community-acquired pneumonia compared to those without lung disease. Conversely, individuals with severe lung disease demanding oxygen therapy exhibit an eightfold greater probability of developing community-acquired pneumonia. The presence of moderate and severe lung disease, as indicated by a predicted percentage forced expiratory volume in one second (FEV1) of 50–80%, has been recognized as a noteworthy risk factor for community-acquired pneumonia (CAP) in older adults aged 65 years and above, in contrast to those possessing healthy or mildly compromised pulmonary function. Moderate COPD exacerbation and hospitalization due to severe COPD exacerbation have also been identified as independent risk factors for CAP in patients with COPD ≥ 45 years of age. The inclusion of patients with these characteristics in study populations is very likely to contribute to the variation in reported pneumonia rates between studies [2,6,8,16,55,70,71,72,73].

Despite the prevalence of evidence-based guidelines pertaining to COPD management, findings from the survey indicate that healthcare professionals display notable deficiencies in their comprehension of essential aspects inherent to the management of this condition. Specifically, at least 50% of surveyed practitioners reported being unaware of established guidelines for both diagnosing and treating COPD [11]. Insufficient awareness of recommended treatment modalities can potentially result in suboptimal management of individuals diagnosed with COPD. Patients receiving COPD treatment are more likely to have more comorbidities, including pneumonia [74] (Figure 2).

### 3.2. Immunodeficiency and Cancer

Patients with compromised immunity exhibit a heightened susceptibility to contracting CAP in comparison to the general populace. This demographic displays an impaired immune system, thereby resulting in a diminished resilience to pathogenic agents. The innate condition of immunosuppression is one possible etiology; nonetheless, the acquired form of immunodeficiency is considerably more prevalent owing to the progression of cancer chemotherapy in recent years. In essence, a strong correlation exists between chronic inflammation and impaired immune response to respiratory pathogens, which promotes the activation of immunosuppressive pathways. This process is believed to be a contributing factor in the pathogenesis of COPD. It is crucial to bear in mind that an immunocompromised individual may harbor multiple concomitant infections.

Some of the following circumstances may contribute to a compromised immune system:

Hematologic malignancies, with the use of corticosteroids and monoclonal antibodies, and T cell dysfunction.

Solid tumors, with high-dose chemotherapy administration, prolonged use of corticosteroids, and marrow transplantation.

Solid organ transplantation, with decreased CD4+ cell counts.

Autoimmune conditions, particularly among individuals receiving immunomodulatory medications such as anti-TNF-α, present a significant clinical concern. Individuals are administered glucocorticoids, as well as other biological and immunomodulatory therapeutic agents for treatment of various medical conditions [75,76,77].

Complications pertaining to human immunodeficiency virus (HIV) primarily affect the lungs, rendering them a crucial target. Observably, individuals with HIV infection are predisposed to a myriad of opportunistic pneumonias, neoplasms, and pulmonary disorders. Opportunistic pneumonias are recognized as the primary causes of morbidity and mortality within the context of pulmonary complications associated with human immunodeficiency virus (HIV). The spectrum of opportunistic pneumonias associated with HIV encompasses a diverse array of pathogens, encompassing bacteria, mycobacteria, fungi, viruses, and parasites.

The prevalence of COPD is higher in people with HIV compared to those without HIV infection. The higher prevalence is explained in part by the higher incidence of smoking in people living with HIV and may be related to an increased susceptibility to respiratory infections, immunosuppressed states, and chronic inflammation [78,79,80,81].

Any type of cancer can directly or indirectly (chemotherapy, hospitalization, metastasis, etc.) lead to pneumonia in COPD, especially in patients with lung cancer or subjects that have been operated upon due to lung cancer. In fact, lung cancer is an important comorbid condition in patients with COPD. The prevalence of lung cancer in COPD is reported to be 2.79%. People with COPD were found to be 6.35 times more likely to have lung cancer than controls. Reduced survival has been reported in patients with lung cancer with comorbidity in the form of COPD [45,46] (Figure 2).

### 3.3. Biomarkers

C-reactive protein (CRP) has been widely used in the management of pneumonia. It is a well-established biomarker of inflammation, but has been regarded as a nonspecific marker in the diagnosis of pneumonia. Nevertheless, it may be of some use in defining the severity of pneumonia [49,50].

Taking into account a baseline eosinophil count of 2% as the threshold value, patients with COPD with lower blood eosinophil counts suffer more pneumonia events than those with higher counts [54].

High blood neutrophil counts in COPD have been associated with an increased risk of pneumonia, independent of ICS use [82].

The neutrophil/lymphocyte ratio (NLR) has been reported to be related to mortality and prognosis in patients with CAP, with better performance as a marker than CRP. The NLR, upon admission to the emergency department, predicts the severity and outcome of CAP with greater prognostic precision than traditional infection markers [83,84,85].

Sphingosine-1-phosphate (S1P) is a bioactive sphingolipid that participates in numerous physiological processes, such as immune responses and maintenance of the endothelial barrier’s integrity. Moreover, sphingosine-1-phosphate (S1P) exhibits potential as a diagnostic and prognostic biomarker for the preliminary evaluation of individuals presenting with pneumonia. The present study indicates a remarkably substantial elevation in the levels of plasma sphingosine-1-phosphate (S1P) in individuals with pneumonia, which also display a positive correlation with the severity of the disease [9,86].

Serum levels of 25-hydroxyvitamin D [25(OH)D] have been shown to be associated with the risk of CAP. Those subjects with 25(OH)D levels < 30 ng/mL had a significantly higher risk of CAP compared to those with levels ≥ 30 ng/mL [87] (Figure 2).

## 4. Chronic Medical Conditions

Compared to patients without COPD, patients with pneumonia with COPD are likely to have more severe pneumonia, with an increase in hospital admissions, and a poorer outcome [18,19,20]. In the first year after the diagnosis of COPD, people are at a 16-fold higher risk of pneumonia compared to patients without COPD. The incidence rate of CAP was found to be 22.4 events per 1000 person-years in the 10 years after the diagnosis of COPD, and more than 50% higher among those classified as having severe COPD [6]. The presence of three or more chronic medical conditions, commonly referred to as multiple morbidity, has been found to be significantly associated with an elevated risk of pneumococcal disease in patients in the age range of 50–64 years. Furthermore, a positive correlation has been established between augmented occurrences of persistent medical conditions and an elevated prevalence of pneumococcal ailment in individuals aged 65 years or above [51].

Individuals afflicted with chronic heart disease (CHD), encompassing congestive heart failure (CHF) and cardiovascular and valve maladies, are vulnerable to contracting CAP with an up to 3.3-fold elevated risk as opposed to those without CHD. Such a risk is contingent upon both the specific condition and advancing age of the afflicted individual. Among individuals aged 65 years or older in the United States, heart disease has been established as a distinct risk factor for CAP, with approximately 16% of total CAP cases being linked to the aforementioned underlying condition.

The risk of CAP was found to be higher in patients with more severe heart disease, while individuals with non-CHF heart disease showed only a modest increase in the risk of CAP compared to those without heart disease. Patients with pneumonia and CHF were found to have an almost two-fold increased risk of hospitalization due to pneumonia compared to those without CHF. The susceptibility to pneumonia was found to be contingent upon both the comorbidities accompanying heart failure and the nature of the administered medical intervention. Individuals diagnosed with cardiomyopathy, as well as those receiving loop diuretic therapy, exhibit a heightened susceptibility to hospitalization resulting from CAP. Amiodarone has been identified as a distinct risk factor for the development of CAP among individuals undergoing treatment for heart failure. Various studies have reported this association, highlighting the need to exercise caution when administering this medication to such patients [16].

Patients with diabetes have an up to 1.4 times higher risk of developing CAP, and the global risk of pneumonia is 1.4-to-4.6-fold higher than in non-diabetic individuals [16].

The prevalence of gastro-esophageal reflux disease (GERD) in individuals with COPD ranges from 17 to 54%. The presence of GERD has been shown to be associated with a higher risk of AECOPD and pneumonia. This is consistent with a defined phenotype for patients with COPD who experience frequent AECOPD (two per year), and with GERD being identified as an independent predictor [55,56,57].

**Figure 3:**
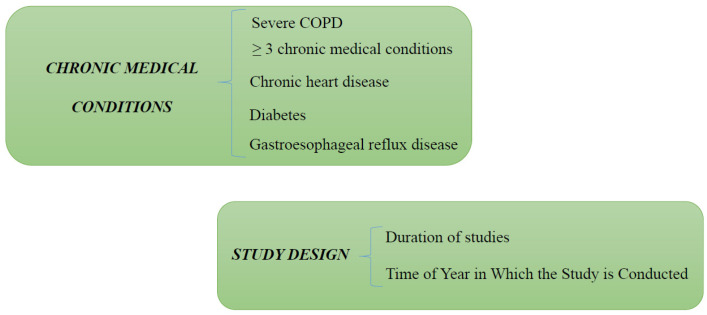
Chronic medical conditions and study design as causes of pneumonia in COPD independent of ICS use.

Pneumococcal disease is often associated with the presence of numerous risk factors in individuals aged 65 years or older, with an estimated 60% exhibiting two or more underlying medical conditions. Numerous conditions have demonstrated a cumulative impact on both the susceptibility of contracting CAP and the associated fatality rate of this pathology. The prevalence of pneumococcal disease escalates proportionately with the amplification of risk factors. The incidence rates are notably elevated for individuals with three or more predisposing conditions [70,88] (Figure 3).

### 4.1. Study Design

The study design can be of great importance in terms of the final results obtained. Furthermore, the duration of studies can be highly variable and, in this regard, the longer the duration, the more likely it is that a pneumonic process develops [89].

The time of year in which the study is conducted can also exert an influence, with more cases of pneumonia recorded in studies that are carried out in winter compared to studies conducted in summer [70].

Elements such as the design of the period prior to starting study treatment are likely to affect the risk that patients develop pneumonia [90].

The absence of randomization in observational studies leads to the risk of confusion by indication [8] (Figure 3).

### 4.2. Triple Therapy

Although the use of triple therapy in COPD appears to have several beneficial parameters, with regard to the risk it represents for these patients due to the possible occurrence of pneumonias, the different studies make the same mistakes described in this review [91,92].

## 5. Conclusions

An increased risk of pneumonia with inhaled corticosteroids in COPD was first described in 2007, and has since been reported in both randomized trials and observational studies. Despite the number of studies reporting this association, many unresolved issues remain regarding the relationship between inhaled corticosteroid use and pneumonia in individuals with COPD.

The idea that the incidence of pneumonia increases in COPD patients treated with ICS, derived from both observational studies and randomized controlled trials, is not supported by firm data, however.

**Figure 4:**
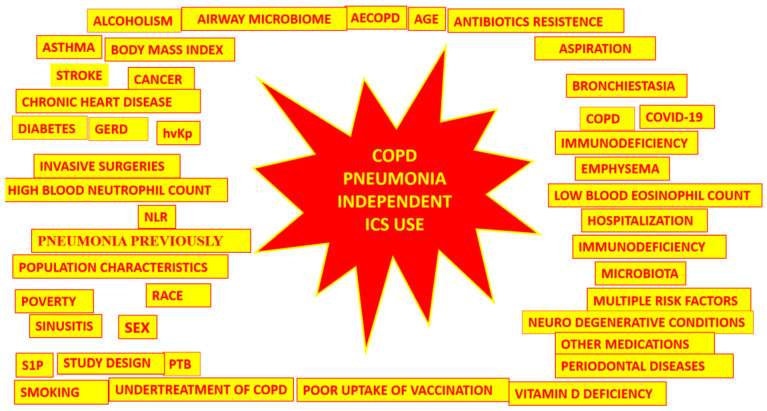
Causes of pneumonia in COPD that are combined and unrelated to ICS usage.

As there is a possibility that the use of ICS in COPD may increase the risk of pneumonia, the elements considered here must be taken into account before prescribing ICS, because they may increase this risk (Figure 4).

## Data Availability

The data presented in this study are available on request from the corresponding author.
